# 'I believe that the staff have reduced their closeness to patients': an exploratory study on the impact of HIV/AIDS on staff in four rural hospitals in Uganda

**DOI:** 10.1186/1472-6963-7-205

**Published:** 2007-12-18

**Authors:** Marjolein Dieleman, Vincent Bwete, Everd Maniple, Mirjam Bakker, Grace Namaganda, John Odaga, Gert Jan van der Wilt

**Affiliations:** 1 KIT Development Policy and Practice, Royal Tropical Institute, Amsterdam, The Netherlands; 2 Faculty of Health Sciences, Uganda Martyrs University, Nkozi, Uganda; 3 KIT Biomedical Research, Royal Tropical Institute, Amsterdam, The Netherlands; 4 Health Partners Uganda Health Cooperation, Kampala, Uganda; 5 Radboud University Medical Centre, Nijmegen, The Netherlands

## Abstract

**Background:**

Staff shortages could harm the provision and quality of health care in Uganda, so staff retention and motivation are crucial. Understanding the impact of HIV/AIDS on staff contributes to designing appropriate retention and motivation strategies. This research aimed 'to identify the influence of HIV/AIDS on staff working in general hospitals at district level in rural areas and to explore support required and offered to deal with HIV/AIDS in the workplace'. Its results were to inform strategies to mitigate the impact of HIV/AIDS on hospital staff.

**Methods:**

A cross-sectional study with qualitative and quantitative components was implemented during two weeks in September 2005. Data were collected in two government and two faith-based private not-for-profit hospitals purposively selected in rural districts in Uganda's Central Region. Researchers interviewed 237 people using a structured questionnaire and held four focus group discussions and 44 in-depth interviews.

**Results:**

HIV/AIDS places both physical and, to some extent, emotional demands on health workers. Eighty-six per cent of respondents reported an increased workload, with 48 per cent regularly working overtime, while 83 per cent feared infection at work, and 36 per cent reported suffering an injury in the previous year. HIV-positive staff remained in hiding, and most staff did not want to get tested as they feared stigmatization. Organizational responses were implemented haphazardly and were limited to providing protective materials and the HIV/AIDS-related services offered to patients. Although most staff felt motivated to work, not being motivated was associated with a lack of daily supervision, a lack of awareness on the availability of HIV/AIDS counselling, using antiretrovirals and working overtime. The specific hospital context influenced staff perceptions and experiences.

**Conclusion:**

HIV/AIDS is a crucially important contextual factor, impacting on working conditions in various ways. Therefore, organizational responses should be integrated into responses to other problematic working conditions and adapted to the local context. Opportunities already exist, such as better use of supervision, educational sessions and staff meetings. However, exchanges on interventions to improve staff motivation and address HIV/AIDS in the health sector are urgently required, including information on results and details of the context and implementation process.

## Background

HIV/AIDS has an impact on health sector workers in various ways. It increases fear of infection at work, changes or increases tasks and workload, and increases the emotional burden and stress levels of health workers [1-3]. However, little concrete evidence exists of the impact of HIV/AIDS on the health sector [4], as few studies have been conducted.

The Ugandan health sector is the main provider of HIV/AIDS-related services for a population of about 29 million people. The HIV prevalence rate is 6.7 per cent among adults (15–49 years) and about 900,000 adults and 110,000 children (0–14 years) live with HIV [5]. About 88 per cent of the Ugandan population live in rural areas [6]. The health sector faces staff shortages. A total of 30,000 health workers were employed in 2004, and yet an extra 5000 qualified staff were required [7]. Most staff are nursing assistants, a cadre with minimal professional health qualifications [7]. A shortage of health workers could negatively influence access to and quality of care. Therefore, retention and motivation of remaining staff is crucial.

Retention of health workers is linked to job satisfaction, which is influenced by various factors such as physical working conditions, relationships with colleagues and managers, pay, promotion, job security, and responsibility, although priorities will differ in different contexts [8]. Job satisfaction influences motivation to work but is not sufficient on its own. When someone is satisfied with his or her job, he/she is not necessarily motivated to perform well. Motivation is defined as 'an individual's degree of willingness to exert and maintain an effort towards organizational goals ([9]: p.1255-6).' Factors that influence motivation to perform well need to be identified in each context. They may include opportunities for promotion or training, opportunities for an increase in remuneration, receiving appreciation for work or obtaining recognition from managers, colleagues and patients.

Published studies about the impact of HIV/AIDS on the health sector and its workers in low-income countries focus mostly on occupational hazards [10,11] and on knowledge, attitudes and practice [12-21]. Some studies focus on a variety of aspects such as stress and burnout, working conditions, knowledge and attitudes, and organizational support [22-26]. Although the studies vary in design and are, therefore, difficult to compare, they confirm that health workers fear infection, face stress and burnout and are concerned about being stigmatized. The main causes identified include a lack of knowledge and skills and a lack of organizational support to deal with fear, stigma, stress and burnout, and changes in tasks and responsibilities.

Studies in Uganda [27-30] have shown that HIV/AIDS influences patient care and that it increases health workers' fear of infection for various reasons. However, a comprehensive overview of the perceptions of managers and health workers of the impact of HIV/AIDS is missing. This is required when designing country-level strategies. Identifying the influence of HIV/AIDS on staff motivation – to design activities that mitigate the impact of HIV/AIDS and integrating these activities in strategies for motivation and retention – is crucial to assure quality of care.

At the time of the study Uganda had 56 districts, served by government, private for-profit and private not-for-profit (PNFP) health facilities. There were 10 regional referral hospitals and 45 district hospitals run by the government. PNFP facilities accounted for 43 per cent of the hospitals and 24 per cent of the lower health care facilities, mostly in rural areas. Many of the PNFP facilities provided health services and trained health workers, and 78 per cent of these facilities were faith based [31].

This article describes the results of a study exploring the effects of HIV/AIDS on hospital staff and organizational responses to mitigate these effects in four different general hospitals in rural districts of the Central Region in Uganda. The study aimed 'to identify the influence of HIV/AIDS on staff working in general hospitals at district level in rural areas and to explore support required and offered to deal with HIV/AIDS at the workplace'.

## Methods

### Research design and research questions

The study design was exploratory and cross-sectional and consisted of a quantitative and a qualitative component. It intended to answer the following research question:

▪ What are the perceptions of hospital managers and staff regarding the effects of HIV/AIDS at their workplace?

▪ What are the current overall working conditions in the selected hospitals?

▪ What are the current support measures offered and required in the selected hospitals to assist staff in dealing with HIV/AIDS at work?

▪ Are hospital staff motivated to work, and to what extent does HIV/AIDS influence staff motivation?

### Theoretical framework

No standard theoretical framework for the impact of HIV/AIDS on hospital staff exists in the literature, therefore the research team developed its own framework. Our main hypothesis was that staff would be discouraged from working because of low motivation and stress, which are influenced by HIV/AIDS and by general working conditions, and that this would contribute to low performance of health systems. According to Chen et al (2004), enhancing the performance of health systems requires, in addition to adequate financial and material resources, workforce objectives on coverage, competence and motivation [32]. Staff motivation is, therefore, an important contributing factor to the performance of health systems. Different theories exist about motivation. In this article health workers' motivation is explained using expectancy theory, adapted to the workplace by Vroom and by Porter and Lawler [33]. This theory describes staff motivation as a rational process of decision making. It explains that staff will be motivated to work if they believe they can perform a task successfully when they put effort into it, if they believe that the outcome will be positive when they perform the task, and if this outcome is valued by them [34]. Our study focused on the impact of HIV/AIDS on motivation. It explored whether staff perceived effects of HIV/AIDS on their work, what these effects were and whether organizational efforts were in place to mitigate this impact, and it identified factors motivating staff to work. Subsequently, during analysis, we tried to identify a relationship between staff motivation and the perceived impact of HIV/AIDS. We also tried to identify if there were differences in perception and responses between the staff of the four hospitals.

### Sampling and study population

Four general hospitals in rural districts were selected purposively, as these were the facilities that provided most HIV/AIDS services close to the population. Purposive sampling is a sampling method used in qualitative research to select 'a limited number of informants strategically so that their in-depth information will give optimal insight into an issue about which little is known ([35]: p.199).' The hospitals were located in four different rural districts in the Central Region in Uganda, a region with a 9.4 per cent HIV prevalence rate, one of the highest in the country [36]. This region consists of four urban and 12 rural districts. In these rural districts comprehensive HIV/AIDS services are provided by 13 public and nine faith-based, private not-for-profit hospitals [oral communication from MoH]. The hospitals were selected according to their type, as different types of hospitals have different working conditions and a different working environment, which might have an impact on staff perceptions and experiences. We were also interested in exploring whether or not individual hospital settings influenced experiences and perceptions, even though hospitals had the same background, so we intended to include more than one hospital of the same type in the sample. Time and budget allowed us to conduct the study in four hospitals: two public and two faith-based. In each health facility, the study population consisted of all health workers, support staff which came in direct contact with patients or patient fluids, and managers.

For the survey, the quantitative component of our study, quota sampling took place, recruiting an appropriate number (quota) in each category of health staff, maintaining a proportional representation of health staff in the sample. Quota sampling means that a quota is set for each attribute (in this case the type of professional cadre), and the quotas are set 'so that they represent together the known distribution of the control attributes across the known population ([37]: p.37).' We intended to include as many health workers as possible, aiming to interview between 30 and 50 per cent of the health workers employed in the selected hospitals. Among support staff cleaners were selected, because they work on the wards and come into contact with patients and with patients' fluids. As they were not the key respondents in the research, we only interviewed a limited number of cleaners. In total, 237 members of staff were recruited according to their availability (presence and having time to be interviewed), from a total of 594 staff employed by the hospitals at the time of the study and in direct contact with patients or patients' fluids. Table 1 shows the distribution of different staff categories interviewed.

**Table 1:** Composition of the survey sample

Type of staff		All hospitals N = 237	Hospital A N = 77	Hospital B N = 46	Hospital C N = 70	Hospital D N = 44
Support staff		36 (15%)	10 (13%)	8 (17%)	12 (17%)	6 (14%)
Clinical staff	Allied health professional	27 (11%)	6 (8%)	5 (11%)	8 (11%)	8 (18%)
	Enrolled Nurse/Midwife	74 (31%)	23 (30%)	14 (30%)	19 (27%)	18 (41%)
	Doctor	8 (3%)	3 (4%)	2 (4%)	1 (1%)	2 (5%)
	Nursing assistant	70 (30%)	28 (36%)	11 (24%)	24 (34%)	7 (16%)
	Registered Nurse/Midwife	22 (9%)	7 (9%)	6 (13%)	6 (9%)	3 (7%)
	Sample of clinical staff as percentage of total number of clinical staff in hospital		65%*	46%	39%	35%

Allied health professionals = clinical officers, laboratory assistants, pharmacy assistants etc.

* The percentage of staff in hospital A participating in the study is higher than in the other hospitals. This is probably due to better mobilization of staff by management compared to the other hospitals.

Respondents for the qualitative component were recruited purposively, using maximum variation sampling. In each hospital, four managers, six health workers from different departments and one or two support staff were selected. Health workers were selected for interviews from among those with experience in working with HIV/AIDS patients, with implementing HIV/AIDS-related tasks and general caring tasks. If a person was not available, either a new appointment was made or another person with similar tasks was selected. Support staff were interviewed according to their availability. In total, 44 respondents were interviewed, and 25 respondents participated in four focus group discussions.

### Data collection

Quantitative data were collected by research assistants who had experience in conducting interviews and who were trained on the background of the study and the research questions. Data were collected through interviews, using a structured questionnaire. Questions were asked about infection control guidelines, availability and use of protective materials, treatment and disposals of sharps, perceived risk of HIV infection at work, occurrence of injuries, and support offered and used to prevent and deal with HIV infection. Additionally, questions were asked about working conditions, supportive supervision, training in HIV/AIDS tasks, and staff motivation. Data were collected during a period of two weeks in September 2005.

Qualitative data were collected by experienced researchers through in-depth interviews and focus group discussions, using a topic guide. Open questions were asked about the impact of HIV/AIDS on work, dealing with HIV/AIDS at work, and support required and offered by the hospital. Questions were also asked about general working conditions and staff motivation.

### Data analysis

Quantitative data were analysed with Epi-info 6.1 and Stata 9.2, using non-parametric tests (Chi-square and Kruskal-Wallis test) for descriptive analyses. Assuming that the members of staff working in a particular hospital were not completely independent, Generalized Estimated Equation (GEE) models were used to determine independent factors associated with not being motivated. Motivation was dichotomized: 'not being motivated' included the categories: indifferent, discouraged and very discouraged, and 'motivated' included the categories motivated and very motivated to the question 'How motivated are you in your work?' Factors associated with not being motivated in univariate analysis (P < 0.10) were selected for multivariate analysis. The final model was created using stepwise backward selection of variables and was checked for confounding. Only the variables that showed a significant effect or acted as confounder were kept in the model. Key variables for analysis included fear of infection, injuries and actions taken, protective materials in place and used, support services to prevent and deal with HIV infection, and general working conditions. Qualitative data were analysed using a framework developed according to the research objectives, key issues and themes. Issues for analysis included the impact of HIV/AIDS at work, dealing with difficulties because of HIV/AIDS (such as fear of infection, stigma, emotions and workload), support required and offered at work, general working conditions, and staff motivation.

### Ethical considerations and quality assurance

The quality of data was safeguarded by using experienced interviewers, assuring the confidentiality and privacy of respondents, and by asking informed consent and permission to tape interviews and focus group discussions. All research instruments were pre-tested. The research team members who conducted the in-depth interviews and focus group discussions were involved in the development of the research protocol and data collection instruments. They also participated in data analysis and report writing. Research assistants were trained to use the structured questionnaire and interviewed under supervision of an experienced researcher.

The validity of data was assured by triangulation. Data were triangulated by:

▪ asking the same questions during focus group discussions and in-depth interviews;

▪ exploring the same topics among support staff, health workers and managers; and

▪ comparing and contrasting information from the interviews and focus group discussions with information from the questionnaires and with the registers and reports from the hospitals.

The protocol was approved by the Ethics Review Committee of Uganda Martyrs University.

## Results

### Characteristics of the study population

All the four hospitals provided services such as health education, voluntary counselling and testing (VCT), antiretroviral (ARV) distribution, treatment of sexually transmitted infections (STIs), treatment of opportunistic infections, and prevention of mother-to-child transmission (PMTCT). The characteristics of each hospital are described in Table 2.

**Table 2:** Main characteristics of each hospital

	Hospital A	Hospital B	Hospital C	Hospital D
Type	Government	Government	PNFP, faith based	PNFP, faith based
No. of beds	100	140	240	120
No. of health staff	103	83	148	108
Bed:staff ratio	1:1.03	1:0.59	1:0.62	1:0.90
New Out-Patient Department cases per day	74	86	85	57
Average length of stay (bed days per patient)	4.75	5.6	5.75	4.3
Patients on Antiretroviral Therapy	95	23	70	68
Observations	-Pilot hospital for Routine Counselling and Testing		-AIDS clinic with 350 outpatients/HIV/AIDS activities financially supported by different donors. -School to train enrolled nurses and laboratory assistants	-Vertical HIV/AIDS programme with own staff attached to the hospital, in addition to hospital HIV/AIDS-related services -School to train enrolled nurses

PNFP = Private Not for Profit

The hospitals differed in number of beds and of clinical staff; public hospital B had the highest bed:staff ratio. The average length of stay for patients in the hospitals varied slightly and was between 4.3 and 5.75 days. There was a large variation in the number of registered ARV patients between the hospitals. Three hospitals had special activities related to HIV/AIDS.

The study sample of the survey in each of the four hospitals was similar in sex and profession (Table 3). In the PNFP hospitals (C and D), staff were significantly younger and had significantly less working experience than staff in the government hospitals (A and B). This corresponds with comments by managers that, after some years of working experience, staff in PNFP hospitals left for better paying public facilities.

**Table 3:** Characteristics of the respondents of the survey

Variables	All hospitals N = 237	Hospital A N = 77	Hospital B N = 46	Hospital C N = 70	Hospital D N = 44	P-value
Sex						0.37
Female (F)	181 (76%)	59 (77%)	32 (70%)	58 (83%)	32 (73%)	
Male (M)	56 (24%)	18 (23%)	14 (30%)	12 (17%)	12 (27%)	
Median age	30	38	33.5	26	28	< 0.001
(Inter Quartile Range)	(25–40)	(29–45)	(26–45)	(23–30)	(24–34)	
Profession						0.45
Highly educated*	57 (24%)	16 (28%)	13 (21%)	15 (30%)	13 (24%)	
Enrolled Nurse-Midwife	74 (31%)	23 (30%)	14 (30%)	19 (27%)	18 (41%)	
Nursing Assistant	70 (30%)	28 (36%)	11 (24%)	24 (34%)	7 (16%)	
Support staff	36 (15%)	10 (13%)	8 (17%)	12 (17%)	6 (14%)	
Years in Hospital						< 0.001
0–4	127 (54%)	28 (36%)	20 (44%)	49 (70%)	30 (68%)	
5–9	34 (14%)	8 (10%)	6 (13%)	8 (11%)	12 (27%)	
10–14	33 (14%)	12 (16%)	9 (20%)	10 (14%)	2 (4.5%)	
15–19	12 (5%)	7 (9%)	3 (7%)	2 (3%)	0	
>19	31 (13%)	22 (29%)	8 (17%)	1 (2%)	0	

*medical officers, registered nurse/midwives and allied health workers such as clinical officer, laboratory assistant, pharmacy assistant etc.

The age range of the managers and the support staff in the study sample of the qualitative component was more or less equal between the different hospitals (Table 4). The health workers' ages and working experience were generally less in the two PNFP hospitals than in the public hospitals (Table 4).

**Table 4:** Characteristics of respondents of the in-depth interviews

Characteristics	Hospital A	Hospital B	Hospital C	Hospital D
**Managers**				
Sex	2 F, 2 M	2 F, 2 M	3 F, 1 M	2 F, 2 M
Age	28, 37, 45, 47	early 30s, 36, 45, 51	early 30s, 33, 60, 29	36, 50, late 50s
Professions	2 medical doctors, 2 registered midwives	2 medical doctors, 2 registered midwives	1 medical doctor, 2 registered nurses, 1 MPH (foreigner)	2 MD, 2 registered nurse/midwives
Work experience	MD: 9, 4 years Midwives: 17, 20 years	MD: 2, 6 years Midwives: 10, 15 years	2 × 1 year, 3, 15 years	MD: 6, 14 years Nurse: 5, 8 years

**Health workers**				
Sex	5 F, 1M	2 F, 4M	2F, 2M	6F
Age	36, 39, 45, 45, 58 (1 missing)	26,48,50,53,57 (1 missing)	21, 24, 26, 30	24, 25,28, 30, 30,55
Profession	1 enrolled nurse/counsellor, 2 enrolled nurse, 1 enrolled midwife, 1 clinical officer and 1 registered nurse	1 enrolled nurse, 1 senior clinical officer, 1 nursing assistant, 2 registered nurses/counsellors, 1 senior anaesthetic officer	1 counsellor, 1 registered nurse, 1 theatre assistant, 1 enrolled nurse	2 enrolled nurses, 1 student midwife, 1 clinical officer, 1 teacher/counsellor, 1 registered nurse
Work experience	6 months to 30 years, 3 around 15 years	6 months to 30 years, 2 around 19 years	2 to 10 years	6 months to 5 years

**Support staff**				
Sex	1 F, 1 M	2 M	1 F	1M
Age	49, 42	21, 32	42	42
Profession	2 cleaners	2 cleaners	cleaner	cleaner
Work experience	19 years (1 missing)	1, 2.5 years	5 years	9 years

Six health workers participated in the focus group discussion in each hospital, except in hospital A where nine people took part. Participants were mostly women, most of whom were enrolled nurses/midwives (17 out of 25 participants). In total only three men participated.

### Perceived effects of AIDS at work

#### Increase in workload

Overall, 86 per cent of the respondents reported that their workload had increased (Table 5). This was lowest in hospital C (74 per cent) and highest in hospital A (94 per cent) (p = 0.01). In hospital A, 70 per cent of respondents reported an increased workload due to extra tasks related to HIV/AIDS, whereas in the other hospitals 26 to 46 per cent of the respondents related an increased workload to staff shortages, extra tasks related to HIV/AIDS or other reasons (p < 0.001). The in-depth interviews in all hospitals revealed that the main reasons for perceived increase in workload were an increase in tasks, especially counselling and ARV provision, an increase in patients, having sicker patients which demand more care and facing staffing shortages. In hospital A, especially the introduction of routine counselling and testing for HV/AIDS was said to contribute to the perceived increase in workload.

**Table 5:** Comparison between hospitals regarding workload, overtime, fear of infection, and injuries *

Variables	All hospitals N = 237	Hospital A N = 77	Hospital B N = 46	Hospital C N = 70	Hospital D N = 44	P
1. Increase in workload	204 (86%)	72 (94%)	42 (91%)	52 (74%)	38 (86%)	0.01
2. Working regularly overtime	114 (48%)	47 (61%)	23 (50%)	25 (36%)	19 (43%)	0.02
3. Afraid of getting infected at work	196 (83%)	64 (83%)	41 (89%)	56 (80%)	35 (79.5%)	0.57
4. Had an injury	85 (36%)	28 (36%)	20 (43%)	19 (27%)	18 (43%)	0.23
5. Reaction after injury:	- Washed the wound	64/81(79%)	17/26 (65%) 2 missing	18/20 (90%)	15/18 (83%) 1 missing	14/17 (82%) 1 missing	0.20
	- Tested the patient	9/81 (11%)	4/26 (15%)	2/20 (10%)	1/18 (6%)	2/17 (12%)	0.78

* = The denominator is given when different from the N

Shortages of qualified staff were mentioned in all hospitals, although records on staff departure demonstrated a low number of staff that had left among government staff: over the past five years in hospitals A and B, respectively, two and seven health workers had left. In the PNFP hospitals staff departure was a lot higher: in hospital D over the past five years 84 health workers had left, whereas the number of staff in hospitals A, B and D was similar (Table 2) and, according to managers, this number had not changed dramatically over the reported years in any of the hospitals. Staff absence during working hours was not mentioned as a major problem, although none of the hospitals registered this systematically.

An average of 48 per cent of respondents reported working overtime regularly (Table 5), which was explained as working outside the normal working schedule. The in-depth interviews showed that working overtime meant that staff skipped breaks, continued working beyond their shift or that staff was called upon by their colleagues when they were free, as illustrated by the following quote:

'*Sometimes they come for me when I am free. It is teamwork, when patients are many you come. If you refuse, they will not help you when you have a problem.' (Health worker)*

Significant differences were found between hospitals in the percentage of staff reporting to work overtime on a regular basis, which was highest in hospital A (61 per cent) and lowest in hospital C (34 per cent) (p = 0.02). In PNFP hospital C, 40 per cent of staff reported that they never worked overtime, corresponding with fewer staff reporting an increase in workload compared to the other hospitals (Table 5). This could be due to the AIDS clinic in hospital C that provided ARV services, counselling and PMTCT services, whereas in the other hospitals these services were integrated.

#### Risk of infection at work

Overall, 83 per cent of the respondents reported being afraid of becoming infected at work. This did not differ significantly between the hospitals (Table 5). Fear of infection was not related to profession, number of years working in the hospital, sex, or having had an injury in the previous year or not. Fear of infection was often reported during the interviews, and some staff answered that they either do not conduct tasks very well, are very cautious to avoid injuries or even avoid tasks involving touching patients known to be HIV-positive or to have AIDS. This is illustrated by the following quote:

'*Health workers fear getting infected with HIV while handling patients. Especially when there are less supplies of protectives in the hospital, this makes health workers so much afraid of contracting HIV. I believe that the staff have reduced their closeness to patients. Now those who are exposed to blood of people, they avoid examinations which will lead to contact with patients' blood.' (Health worker)*

Injuries were common in all hospitals: 36 per cent of the respondents reported an injury in the last year (Table 5), and in all in-depth interviews staff mentioned either having had an injury or knowing someone who had had a needle-stick injury. According to the interviews, the most common reactions after injuries in all hospitals was to wash the wound. This was confirmed by most of the survey respondents (79 per cent). A quote by a health worker in a focus group discussion illustrates this:

'*I was pricked by a needle when I was putting an IV-line, I ran water on the finger to let the blood flow on it. I asked the patient to tell me about herself and she told me the husband had died. I just prayed to God*'.

On very few occasions (11 per cent) the patient was tested, and only one person went for post-exposure prophylaxis (PEP), in hospital A.

#### Emotions

During the interviews most respondents said they accepted HIV/AIDS patients as people needing a lot of care and AIDS as 'any other disease'. Although staff did not lose morale, they were at times affected and felt frustrated, sad or depressed, especially when poor patients were left alone by their carers and when patients did not improve despite treatment. As one respondent stated:

'*No, it doesn't affect me and for others, it is difficult to know because we haven't talked about it. I see colleagues being very concerned about the patients and giving their best. We have a doctor who gives patients money.' (Health worker)*

There were no major differences in answers between the hospitals. In most hospitals health workers and support staff also acknowledged being personally affected. Emotions related to providing care were difficult to discuss. This might be because staff did not think about emotional stress and saw HIV/AIDS as part of normal life, or because staff and management were not used to discussing feelings related to work.

#### HIV-positive colleagues

Discussing the status of colleagues or staff themselves was very difficult. Staff and management reported that hospital staff did not want to be tested and come out in the open due to fear of being stigmatized and, as a result, being isolated and talked about by their colleagues and by patients.

'*The staff themselves fear stigma. The other staff are not so sympathetic. No. There was a midwife who died last year, she was being abused by another midwife. They were on duty and they got some quarrel over a patient and one of them told the other that "you know you are sick and tomorrow you are going to die" and I was there. And that is the thing that is making staff fear to test.' (Health worker)*

HIV-positive colleagues were mainly suspected through clinical signs, and they were suspected if they had lost their partner due to AIDS. Staff reported that HIV-positive colleagues were tested and treated elsewhere and not in the hospital where they work. AIDS-related deaths among staff occurred in all hospitals, although limited numbers were reported. In hospital A, B, C and D over the past five years, respectively four, six, seven and three staff were reported to have died from AIDS-related illness.

### Organizational responses

#### Dealing with workload

Lack of qualified staff was a problem for all hospitals. In hospitals A, B and C most staff (63 to 70 per cent) were enrolled nurses and nursing assistants. In hospital D, only 40 per cent of staff were enrolled nurses and nursing assistants. It is likely that this hospital used students to do certain types of work. The problem of a limited number of qualified staff was addressed by having tasks conducted by less- or non-qualified staff, or qualified staff were asked to work overtime. According to respondents of the in-depth interviews, both ways of addressing the problem risked having a negative impact on quality of care, as illustrated by the following quote:

'*Yes (it influences quality), counselling may not be perfect because they have to combine it with other work in the ward, which is also not perfected.' (Manager)*

When working overtime, 81 per cent overall received no compensation (Table 6). In hospital C, this was much lower (38 per cent) than the other hospitals, because overtime mainly occurred in the AIDS clinic where it was paid.

**Table 6:** Comparison between hospitals for various variables on staff motivation and organizational responses.

Variables	All hospitals N = 237	Hospital A N = 77	Hospital B N = 46	Hospital C N = 70	Hospital D N = 44	P
1. Staff motivation						
- Staff motivated	168 (71%)	58 (75%)	26 (57%)	54 (77%)	30 (68%)	
- Staff indifferent	32 (14%)	13 (17%)	11 (24%)	4 (6%)	4 (9%)	
- Staff discouraged	37 (16%)	6 (8%)	20% (9)	12 (17%)	10 (23%)	0.02
2. NOT compensated for overtime	141/174 (81%)	58/67, 1 missing (88%)	33/35 (94%)	16/42 (38%)	34/44 (77%)	< 0.001
3. Feels adequately protected	142/236 (60%)	43 (56%)	26 (57%)	40 (70%)	24, 1 missing (56%)	0.26
4. Knows how to protect him/herself	223 (94%)	72 (94%)	41 (89%)	67 (96%)	43 (98%)	0.33
5. Guidelines for protection						
-Awareness	183 (77%)	68 (88%)	35 (76%)	52 (74%)	28 (64%)	0.02
-Available	92/183 (50%)	42/68 (62%)	12/35 (34%)	24/52 (46%)	14/28 (50%)	0.23
6. Sterilizing metallic instruments**						
- Autoclaving	159 (84%)	45 (69%)	27 (79%)	53 (95%)	34 (100%)	<0.001
- Boiling	89 (47%)	57 (88%)	13 (38%)	12 (21%)	7 (21%)	<0.001
- Chemicals	64 (34%)	36 (55%)	1 (3%)	6 (11%)	21 (62%)	<0.001
7. Protective gear always available:						
- Gloves	205 (88%)	69 (90%)	39 (85%)	65 (94%)	32 (76%)	0.04
- Antiseptics***	185 (93%)	59 (88%)	36 (95%)	56 (97%)	34 (94%)	0.27
- Apron**	156 (78%)	48 (72%)	28 (74%)	49 (85%)	31 (86%)	0.01
- Gumboots	130 (55%)	41 (53%)	21 (46%)	47 (67%)	21 (50%)	0.01
- Vacutainers**	66 (33%)	29 (43%)	3 (8%)	21 (36%)	13 (36%)	<0.001
- Masks**	76 (38%)	22 (33%)	8 (21%)	26 (45%)	20 (56%)	< 0.001
- Goggles**	23 (12%)	3 (5%)	2 (5%)	14 (24%)	4 (11%)	0.02
8. Awareness and accessibility of PEP:						
- Is aware of PEP	130 (55%)	60 (78%)	16 (35%)	27 (39%)	27 (61%)	<0.001
- Believes that PEP is offered in the hospital	71 (30%)	50 (65%)	3 (6.5%)	11 (16%)	7 (16%)	<0.001
9. Availability of HIV/AIDS-related services for staff						
- Condoms	159 (67%)	74 (96%)	39 (85%)	45 (64%)	1 (2%)	<0.001
- Counselling	230 (90%)	77 (100%)	40 (87%)	66 (94%)	30 (68%)	<0.001
- HIV Testing	217 (92%)	77 (100%)	41 (89%)	66 (94%)	33 (75%)	<0.001
- Antiretrovirals	192 (81%)	72 (94%)	33 (72%)	56 (80%)	31 (71%)	0.03
- Health Education	217 (92%)	73 (95%)	40 (87%)	66 (94%)	38 (86%)	0.28

* = The denominator is given when different from the N

** = Selection of people to whom the particular question applied, excluding 2 persons who did not use any sterilizing method, N = 189

*** = only those persons were selected who needed to use these measures in their work

PEP = Post-Exposure Prophylaxis

#### Protection against HIV infection

Although the majority of respondents (60 per cent) reported feeling adequately protected (Table 6), 77 per cent of these were still afraid of getting infected. Existence of fear despite access to protective materials was confirmed during the in-depth interviews and is illustrated by the following quote:

'*We use the gloves, these masks, gumboots and aprons. But we still feel we are not secure. We use 2–3 gloves and we end up using a lot of gloves, more than the required amount.' (Health worker at a maternity ward)*

Managers and staff reported that at times protective material was of poor quality, contributing to fear of infection.

There were no significant differences in feeling adequately protected between hospitals, professional cadres, duration of service in hospital or having had a needle-stick injury in the previous year or not. Significantly more men than women felt adequately protected.

Most staff (94 per cent) reported knowing how to protect themselves (Table 6). Overall, 77 per cent said they were aware of infection control guidelines, although this differed significantly between hospitals, varying from 64 per cent in hospital D to 88 per cent in hospital A. In general, 73 per cent of the respondents reported being informed about such guidelines, and on average 50 per cent said that guidelines were available (Table 6).

In all hospitals, between 96 and 100 per cent of staff reported that they use disposable needles, and 97 to 100 per cent reported using safety boxes for disposal of these needles. In addition, 82 per cent of staff using needles reported that used needles were burned (Table 6). Respondents in hospitals C and D reported using autoclaving for sterilizing equipment significantly more often than respondents in hospital A and B (Table 6). In hospital A, 88 per cent of staff reported boiling metallic instruments.

Various protective materials were reported to be available, as shown in Table 6. Hospital B in particular lacked materials, as significantly fewer staff reported the availability of five types of protective materials (aprons, gumboots, vacutainers, masks and goggles). In this hospital staff reported that when stock outs occurred at times patients were asked to buy gloves. Gloves and antiseptics were used most of the time in all four hospitals. In hospital D significantly more respondents (91 per cent) used aprons, probably due to availability. Other materials in the four hospitals were used to a lesser extent, as their availability was limited.

Staff need to be aware of the possibilities of getting PEP after an injury. In hospitals A and D significantly more staff were aware of PEP compared to hospitals B and C (Table 6). There was a significant difference in perceptions of accessibility to PEP among staff in hospital A compared to the staff in the other hospitals. However, in total only two people reported ever having used PEP.

#### Training

Overall, 64 per cent of health workers who answered as being responsible for tasks related to HIV/AIDS were responsible for 5–10 different tasks. Tasks that were reported by more than 50 per cent of the respondents were health education, counseling, STI treatment and caring for HIV/AIDS patients on the wards. In hospital C, significantly more people reported having only one task related to HIV/AIDS, which might be due to the existence of an AIDS clinic. In hospital A, significantly more staff reported being involved in all tasks, which is likely to be due to the introduction of routine counselling and testing (RCT). An average of 50 per cent of the respondents reported being trained for all their HIV/AIDS tasks, ranging from 46 per cent in hospital B to 56 per cent in hospital D (data not shown). Tasks in which fewer than 20 per cent of respondents reported being trained were treatment of opportunistic infections, STI treatment, training and supervision of carers, and caring for HIV/AIDS patients on the wards. In the interviews, most staff and managers answered that especially training in testing and counselling was organized.

#### Coping emotionally

Responses to feelings of frustration, depression and sadness were similar in the four hospitals: hospital staff reported 'doing their best' and accepting it when they were not able to help, either ignoring the feeling, dealing with it by themselves informally by praying, or talking to colleagues. At times they did consult seniors when problems occurred, as illustrated by this quote:

'*My staff complain, they say: we have lost a patient and we did not want this patient to die. I tell them that is part of life, we have done our best and maybe it was God's plan.' (Manager of a ward)*

In none of the four hospitals was a system in place to assist staff when they faced emotional difficulties, although opportunities existed. Three hospitals organized regular educational sessions, in which HIV/AIDS-related topics were discussed. In hospital B such sessions were less regular. However, these sessions might be considered too 'public' to deal with emotions. Supervision might offer another opportunity for those in charge to discuss difficulties in dealing with HIV/AIDS patients. Overall 57 per cent of respondents reported being supervised daily and 27 per cent at least monthly. Daily supervision was lowest in hospital B (39 per cent) and highest in hospital A (68 per cent) (p = 0.02). Supervision was considered by most respondents in the hospitals sufficient to cope with work (91 per cent in hospital A, 76–77 per cent in hospitals B, C and D, NS). The PNFP hospitals offered spiritual guidance: hospital C had early-morning devotion times, in which problems were sometimes discussed, and hospital D offered a minister for talking and praying. In the in-depth interviews there were no clear differences in answers between staff in the different hospitals, which indicates that the spiritual guidance provided did not make it noticeably easier to cope with HIV/AIDS patients.

#### Support offered to deal with HIV/AIDS at work

Staff could make use of the available HIV/AIDS services, although respondents reported differently with respect to their availability (Table 6). There were significant differences in the reported use of services by staff between the hospitals: in hospital A, 83 per cent reported using counselling and testing services, compared to an average of respectively 61 per cent and 63 per cent (p < 0.001). This is likely to be linked to the introduction of RCT whereby health workers are able to test themselves. In the PNFP hospitals more staff reported having used testing services than counselling services. For hospital C this might mean that staff tested themselves secretly; for hospital D it might mean that they tested using private providers, as the use of testing kits had to be reported. Overall, only a limited number of staff reported using ARVs: about 81 per cent of respondents knew ARVs were available, 63 per cent reported having tested, and out of all of those only 5 per cent reported using ARVs.

None of the hospitals in the survey had a written policy to deal with HIV-positive staff. Respondents in all hospitals reported that HIV-positive staff continued working until they were too ill, and that they did not come out in the open. Staff known by management to be HIV-positive received free treatment: ARVs when meeting the requirements, treatment of opportunistic infections, and counselling. In addition, respondents reported that hospital staff could be given lighter duties and private rooms in the hospital for admission. Staff and management in the public hospitals reported that salaries were paid until death of a sick staff member. In the PNFP hospitals salaries were reported as being paid up to three months, although most staff were not sure about this. Only in hospital C did staff report that financial support was received. Support to HIV-positive staff proposed by respondents in all hospitals was food, financial support to staff and relatives, lighter duties, and private rooms.

### Staff motivation to work

When asked about motivation in general, most staff (71 per cent) in the four hospitals reported feeling motivated (Table 6). Motivation among respondents ranged between 77 per cent for hospital C and 57 per cent for hospital B (Table 6). For staff in hospitals A, C and D the main reasons for being motivated to work reported in the survey were 'liking the job', followed by pay (extra pay or salary increase). In hospital B the main reason for motivation was pay, followed by liking the job. In hospital A, only 8 per cent of the respondents reported feeling discouraged from working – significantly less than the other hospitals (p = 0.02) – the main reason being workload. In hospitals B, C and D the main reason for discouragement was poor pay. In the in-depth interviews staff also answered being motivated for their work, with the most important motivating factor being 'liking the work'. Other reasons for motivation that were mentioned in hospitals A, C and D were supervision, supportive management, teamwork, and training opportunities, whereas in hospital B pay was mentioned as an important motivating factor. A salient finding from the in-depth interviews was the importance that staff gave to support from colleagues and management, through communication, team work and supervision.

'*If you have a problem, there is someone to talk to – there is good communication.' (Health worker, hospital A)*

Results of the multivariate analysis showed that not being motivated was associated with having a high or medium level of education, being male, working overtime, not being aware of counselling or believing it is not offered, using ARVs, and not receiving immediate supervision on a daily basis (Table 7).

**Table 7:** Association between independent variables and staff motivation as revealed by multivariate analyses, using Generalized Estimated Equation (GEE)

	N	% not motivated	Adjusted OR (95% Confidence Interval)
Profession			
Support	36	8 (22%)	0.57 (0.09–3.44)
Low education (Nursing assistant)	70	13 (19%)	1
Mid and high education (Enrolled nurse, Registered nurse, Allied health professional)	131	48 (37%)	1.90 (1.35–2.67)
Sex			
Female	181	49 (27%)	1
Male	56	20 (36%)	1.61 (1.26–2.08)
Perceived risk of HIV			
Not afraid of getting infected at work	41	8 (19.5%)	1
Afraid of getting infected at work, but feels adequately protected	110	37 (36.5%)	2.75 (0.89–8.54)
Afraid of getting infected at work and does not feel adequately protected	86	24 (28%)	1.41 (0.73–2.74)
Works overtime			
Yes	175	62 (35%)	4.21 (2.93–6.05)
No	62	7 (11%)	1
Provision of counselling			
Yes	213	55 (26%)	1
No or does not know	24	14 (58%)	4.77 (3.52–6.45)
Use of Antiretrovirals			
Yes	11	5 (45.5%)	2.05 (1.15–3.64)
No	226	64 (28%)	1
Frequency of immediate supervision			
Every day	136	28 (21%)	1
Less than every day	101	41 (41%)	2.54 (1.68–3.84)

OR = Odds Ratio

## Discussion

In this study staff and managers reported that HIV/AIDS has an impact on workload, leads to changes in tasks and affects emotions, although the latter was less pronounced in this study. Injuries were reported to be common, and most staff feared infection at work. Respondents knew colleagues who were HIV-positive, although HIV-positive staff remained in hiding, and staff did not want to get tested due to fear of being stigmatized. No HIV-positive staff talked openly about their HIV status. The reported impact of HIV/AIDS demonstrated in these Ugandan hospitals corroborates published studies elsewhere: frequent occurrence of injuries, reported by 36 per cent of respondents in our study, is reported by 57 per cent of respondents at the central hospital in Uganda [29], and ranged between 26 and 53 per cent in studies reporting on injuries elsewhere [22,24,26]. Fear of infection, in our study reported by 83 per cent of respondents, varied in two Ugandan studies between 30 and 47 per cent [27,28], and our study corroborates reported fear of infection from studies elsewhere [12,22,24,26]. In our study 77 per cent of those respondents who felt adequately protected feared getting infected. These findings corroborate two studies among doctors in Nigeria, which show that feelings of fear of infection persist, despite the availability and use of protective materials [38,39]. A number of studies in other countries with a high HIV prevalence rate demonstrated that staff felt stressed and faced burnout, often being emotionally exhausted [14,22-26]. This was less pronounced in Uganda. Studies in Zambia [22,26] reported, as in our study, that HIV-positive staff are not willing to tell others about their status and that health workers in general are unwilling to be tested.

In the Ugandan hospitals, organizational responses to the impact of HIV/AIDS were implemented haphazardly. None of the hospitals had written policies to prevent and mitigate the impact of HIV/AIDS and to support HIV-positive staff. Organizational responses were reported to consist of informing staff about infection control, making protective materials and existing HIV/AIDS-related services available, although in none of the hospitals respondents reported that these services were clearly communicated to staff. Areas that were not explicitly addressed in any of the hospitals were stigma, HIV counselling and testing among staff, supporting HIV-positive staff, availability and use of PEP, and emotional support. Lack of organizational support is also shown in studies in Zambia [22,26]. The findings show that management needs to urgently address the impact of HIV/AIDS in health facilities. Workplace HIV/AIDS policies need to be designed and implemented, and use could be made of the generic guidelines developed by ILO/WHO [40] and of workplace policies that have been designed for the private sector in Uganda [41].

We explored the relationship between the perceived impact of HIV/AIDS and staff motivation. Motivation appeared to be determined, among others, by working conditions, such as overtime, frequency of supervision, provision of HIV counselling, and use of ARVs by staff. These factors are greatly influenced by HIV/AIDS. It might be that because of a lack of strategies to support HIV-positive staff, staff using ARVs do not feel motivated. This could not be explored, as no HIV-positive staff came forward during our study.

Staff with a higher level of education in particular were less often motivated than other staff, which might be linked to the reported lack of qualified staff and, therefore, having more responsibilities. Men are less often motivated than women in the survey, but it is not clear why, and answers from the interviews did not confirm this.

The most important reported reason for staff motivation was 'liking the work', and salaries and financial benefits appeared less important. This is corroborated elsewhere, and various studies [42-47] show that, although financial incentives are important, other motivating factors were appreciation, recognition and career possibilities. A number of reported reasons for motivation, such as 'liking the work', 'recognition', 'teamwork', and 'salaries and financial benefits' were not included in the multivariate analysis, as no separate questions were asked with respect to these variables. These could have been determinants or confounders for staff motivation and would need to be included in further studies.

As low motivation of health workers contributes to poor health worker performance and thus affects quality of care, Human Resources Management (HRM) activities to improve staff motivation need to be implemented. Managers should be aware that there is a complicated relationship between motivation and performance. According to expectancy theory, motivation to perform is a combination of feeling able to successfully perform a task when putting effort into it, obtaining a positive outcome (reward) upon completion, and valuing this outcome. This means that health facility managers need to implement HRM activities and use leadership skills to:

▪ assure that the expected level of performance is discussed and agreed upon by staff and management;

▪ support staff in such a way that they feel able to achieve the expected level of performance;

▪ assure that expected positive outcomes of performance (eg financial or non-financial rewards) outweigh expected negative outcomes (e.g. being tired and overworked); and

▪ assure that expected rewards are provided when performance is achieved [48].

A combination of interventions in all these areas is likely to lead to motivation for performance.

Our study identified that staff and managers considered HIV/AIDS to be constraining their work, as it either led to a perceived negative outcome (such as fear of getting infected while delivering care) or had an impact on their perceived ability to provide quality care (due to increased workload, emotional stress, changes in tasks and limited training in new tasks). Integrating activities to prevent and mitigate the impact of HIV/AIDS into existing HRM activities, instead of developing a 'vertical' HIV/AIDS workplace programme, can improve these perceptions. Examples of this type of activity are the integration of discussions on infection control, stigma and difficulties with HIV/AIDS patients into staff meetings and daily supervision; including HIV/AIDS-related topics in educational sessions to improve staff knowledge and skills; and including support to HIV-positive staff in workplace policies for chronically ill staff. Workload issues can be addressed by improving teamwork, rotating tasks, and taking measures aimed at staff attraction and retention. Our study identified motivating and discouraging factors among staff, but ranking these factors is required to assist managers to prioritize and align incentives for performance with valued positive outcomes of staff performance.

Caution has to be taken to replicate strategies without adapting these to the prevailing context. Although the type of hospital (public or PNFP) did not influence the reported impact of HIV/AIDS or organizational responses, the specific hospital context did seem to influence the perceptions and experiences of hospital staff, although differences were not always statistically significant. Two examples to illustrate this (Table 5 and 6):

In hospital B, staff were generally less motivated. The reported working conditions were less positive than in the other hospitals: hospital B had the highest bed:staff ratio, the lowest availability of five types of protective materials, the lowest number of staff that received daily supervision, irregularly organized educational sessions and the highest number of staff reporting not receiving compensation for overtime. In addition, it was one of the two hospitals with the highest number of injuries, and respondents of hospital B had the lowest knowledge of PEP and its availability in the hospital. Lastly, although pay was higher in hospital B than in hospitals C and D, staff motivation in hospital B was lower than hospitals C and D. A focus for interventions would be to analyse leadership and management and to improve upon available activities such as supervision and educational sessions.

On the other hand, in hospital A many respondents were motivated despite the fact that the most overtime was reported in this hospital and a low number of staff received compensation. Hospital A had a high number of respondents being aware of guidelines, knowing about and using HIV/AIDS-related services for staff and being aware of PEP offered in the hospital. In this hospital a high number of respondents reported being supervised daily. However, in hospital A staff complained about a heavy workload due to extra tasks related to HIV/AIDS. This is in contradiction to the bed:staff ratio and the reported number of new patients in the outpatient department and might be linked to reported extra tasks related to HIV/AIDS such as RCT. The focus for intervention might be on evaluating staff experiences with RCT so as to better adapt these to staff capabilities.

It is important to know that although general measures should be designed to mitigate the impact of HIV/AIDS and to motivate staff, these examples demonstrate that each hospital management team needs to have a different focus in strategies. They show that differences occur in leadership and management, availability of resources and organization of services between hospitals working in similar conditions. Management needs skills and support to analyse the working conditions in their facilities and to adapt generic guidelines to their own specific situation.

Informing policymakers which interventions are successful under which circumstances and for which staff groups is important, as it allows the formulation and implementation of evidence-based approaches [49]. Various authors [50,51] describe the importance of taking the context and process into consideration when formulating and implementing interventions to address performance problems. Identifying strategies to address factors contributing to performance problems, such as low motivation, is important but managers need to be aware that blue print solutions do not exist. Health systems are social systems which are open and thus are influenced by and interact with their context. Additionally, the way interventions are implemented depends on the vision, skills and experiences of stakeholders involved in its implementation (management, health workers, support staff, district teams etc). This is also the case for strategies for staff motivation in the health sector. Therefore, evidence building needs to include information on the process of implementation, the context and any changes in the context. Randomized trials, which for health system interventions are considered by many the most credible designs for evidence building [50], normally do not include data collection on context and process. To answer the question 'what works for whom and why', these trials need to be complemented with different types of data and of data collection methods, such as methods to describe practitioners' views on lessons learned and conditions for success. Up to now little has been written on what works and what does not with respect to staff performance, their motivation and retention in the health sector in low-income countries (among others, [52,53]). Experiences with activities to mitigate the impact of HIV/AIDS and the integration of such activities into motivation and retention strategies for health workers are remarkably scarce in literature. There is an urgent need to document and share experiences with interventions to motivate and retain staff in low-income countries and with activities mitigating the impact of HIV/AIDS on the health sector and its workers.

### Study limitations

We were not able to measure stress, as no validated instrument for Uganda exists. Therefore, the influence of HIV/AIDS on stress could not be identified. Additionally, for some members of the research team it was difficult to probe on sensitive topics such as personal experiences with HIV/AIDS, HIV status of respondents and colleagues, and emotional feelings, which might have influenced data collection. The survey was based on the availability of staff, which could have caused a selection bias and which we were not able to check as data on absence and sick leave were not available in the hospitals. However, management assured us that absence and sick leave were not major problems faced in any of the hospitals and that staff were systematically scheduled to work in different shifts, without differences in profile. In addition, our own impression during the study was that absence due to stress, burnout or low motivation was not an issue in any of the hospitals. The questionnaire and the interview guide could have better addressed motivation by including questions in line with expectancy theory. Lastly, the results reflect staff opinion on their knowledge, skills and practice. Confirming if reported knowledge, skills and practice correspond with actual knowledge, skills and practice was not possible, due to time and budget constraints.

## Conclusion

The study demonstrates that HIV/AIDS is a crucially important contextual factor, impacting on working conditions and staff motivation in various ways, and that staff perceptions and experiences with HIV/AIDS are influenced by individual hospital settings.

Given the fact that HIV/AIDS is a contextual factor, exacerbating working conditions that are already difficult, organizational responses to address the impact of HIV/AIDS need to be integrated with responses to address other problematic working conditions. Opportunities are present such as supervision, educational sessions, staff meetings and clearly providing counsellors to hospital staff. However, this can only be achieved if HIV/AIDS workplace policy and programmes are systematically developed and implemented, and when they are adapted to the local context. More information exchange on successes and failures of interventions to improve staff motivation and address HIV/AIDS in the health sector is urgently required to assure appropriate resource allocation. This requires additional data collection methods to the commonly applied randomized trials, which often exclude the context and process of implementation.

## Competing interests

The author(s) declare that they have no competing interests.

## Authors' contributions

MD and VB were principal investigators of the study and were responsible for protocol development, study implementation, data analysis, and report writing. MD drafted the manuscript. EM, GN and OJ were members of the research team and contributed to protocol development, study implementation, data analysis, and report writing. VB, EM, GN and OJ commented on the first manuscript. MB analysed data of the quantitative study component, contributed to report writing and commented on the draft manuscripts. GvdW discussed the conceptual framework for the study and substantially commented on the draft manuscripts. All authors read and approved the final manuscript.

## Pre-publication history

The pre-publication history for this paper can be accessed here:


